# Supplementary material for: Interbacterial competition and anti‐predatory behaviour of environmental Vibrio cholerae strains
Source: Environ Microbiol. 2020 Oct 2;22(10):4485–504. doi: 10.1111/1462-2920.15224 (PMC7702109; doi:10.1111/1462-2920.15224)
Supplement: Supplementary file 1 — Table S1 Vibrio cholerae, Escherichia coli and Klebsiella pneumoniae strains used in this study. Table S2. Information of the long‐read whole genome sequencing data and assemblies of NCBI BioProject PRJNA633476. Table S3. BlastP‐predicted T6SS effector and immunity proteins1 of the environmental V. cholerae isolates. Table S4. Matrices of % identity (percentage of residues which are identical) among VgrG1‐ACD proteins in T6SS auxiliary cluster 1 from clade C and D environmental V. cholerae strains and pandemic strain A1552. Table S5. Matrices of % identity (percentage of residues that are identical) among the effector and immunity proteins harboured in the T6SS large cluster from the environmental V. cholerae strains and the pandemic strain A1552. Table S6. Matrices of % identity (percentage of residues that are identical) among the effector and immunity proteins harboured in the T6SS auxiliary cluster 1 from the environmental V. cholerae strains and the pandemic strain A1552. Table S7. Matrices of % identity (percentage of residues that are identical) among the effector and immunity proteins harboured in the T6SS auxiliary cluster 2 from the environmental V. cholerae strains and the pandemic strain A1552. [file EMI-22-4485-s001.pdf]

# Supporting Information

## Interbacterial competition and anti-predatory behavior of environmental *Vibrio cholerae* strains

**Natália C. Drebes Dörr and Melanie Blokesch***

### Supporting tables and legends

**Table S1.** *Vibrio cholerae*, *Escherichia coli* and *Klebsiella pneumoniae* strains used in this study.

| Strain names | Genotype / description | Internal strain number | Reference |
|---|---|---|---|
| ***V. cholerae*** | | | |
| A1552 (WT) | Wild-type, O1 El Tor Inaba; Rif$^R$ | MB_1 | (Yildiz and Schoolnik, 1998) |
| W10G | Environmental isolate (clade A) collected in Waddell Creek (CA, USA) in October 2004 | MB_5537 | (Keymer *et al.*, 2007) |
| SA3G | Environmental isolate (clade B) collected in Old Salinas River (CA, USA) in March 2004 | MB_957 | (Keymer *et al.*, 2007) |
| SA5Y | Environmental isolate (clade B) collected in Old Salinas River (CA, USA) in May 2004 | MB_353 | (Keymer *et al.*, 2007) |
| SL4G | Environmental isolate (clade B) collected in San Lorenzo River (CA, USA) in April 2004 | MB_955 | (Keymer *et al.*, 2007) |
| SL5Y | Environmental isolate (clade B) collected in San Lorenzo River (CA, USA) in May 2004 | MB_954 | (Keymer *et al.*, 2007) |
| SO5Y | Environmental isolate (clade B) collected in Soquel Creek (CA, USA) in May 2004 | MB_960 | (Keymer *et al.*, 2007) |
| L6G | Environmental isolate (clade C) collected in Lagunitas Creek (CA, USA) in June 2004 | MB_956 | (Keymer *et al.*, 2007) |
| SL6Y | Environmental isolate (clade C) collected in San Lorenzo River (CA, USA) in June 2004 | MB_953 | (Keymer *et al.*, 2007) |
| SP6G | Environmental isolate (clade C) collected in San Pedro Creek (CA, USA) in June 2004 | MB_964 | (Keymer *et al.*, 2007) |
| SP7G | Environmental isolate (clade C) collected in San Pedro Creek (CA, USA) in July 2004 | MB_952 | (Keymer *et al.*, 2007) |
| W6G | Environmental isolate (clade C) collected in Waddell Creek (CA, USA) in June 2004 | MB_354 | (Keymer *et al.*, 2007) |
| W7G | Environmental isolate (clade C) collected in Waddell Creek (CA, USA) in July 2004 | MB_962 | (Keymer *et al.*, 2007) |
| E7G | Environmental isolate (clade D) collected in Moss Landing Harbor (CA, USA) in July 2004 | MB_963 | (Keymer *et al.*, 2007) |
| SA7G | Environmental isolate (clade D) collected in Old Salinas River (CA, USA) in July 2004 | MB_959 | (Keymer *et al.*, 2007) |
| SA10G | Environmental isolate (clade D) collected in Old Salinas River (CA, USA) in October 2004 | MB_5539 | (Keymer *et al.*, 2007) |
| A1552Δ*hlyA* | A1552 with *aph* cassette in *hlyA* (A1552VC_A02993; homolog to VCA0219*); Rif$^R$, Kan$^R$ | MB_3934 | This study |
| W10GΔ*hlyA* | W10G with *aph* cassette in *hlyA* (HPY12_14900); Kan$^R$ | MB_8548 | This study |
| SA5YΔ*hlyA* | SA5Y with *aph* cassette in *hlyA* (VC-Sa5Y_02920); Kan$^R$ | MB_4622 | This study |
| SL6YΔ*hlyA* | SL6Y with *cat* cassette in *hlyA* (HPY06_14140); Cm$^R$ | MB_8549 | This study |
| SA10GΔ*hlyA* | SA10G with *aph* cassette in *hlyA* (HPY11_14585); Kan$^R$ | MB_8550 | This study |
| A1552Δ*vipA* | A1552 with *aph* cassette in *vipA* (A1552VC_A02892; homolog to VCA0107); Rif$^R$, Kan$^R$ | MB_3012 | This study |
| W10GΔ*vipA* | W10G with *aph* cassette in *vipA* (HPY12_14395); Kan$^R$ | MB_6850 | This study |
| SA5YΔ*vipA* | SA5Y with *aph* cassette in *vipA* (VC-Sa5Y_02822); Kan$^R$ | MB_5772 | This study |
| L6GΔ*vipA* | L6G with *aph* cassette in *vipA* (HPY16_13230); Kan$^R$ | MB_8628 | This study |
| SL6YΔ*vipA* | SL6Y with *aph* cassette in *vipA* (HPY06_13635); Kan$^R$ | MB_8551 | This study |
| SP7GΔ*vipA* | SP7G with *aph* cassette in *vipA* (HPY08_13415); Kan$^R$ | MB_8629 | This study |
| SP7GΔ*vipA*-Rif$^R$ | SP7G with *aph* cassette in *vipA* (HPY08_13415); transformed with PCR of *rpoB* gene containing mutation (S531F) that confers rifampicin resistance; Kan$^R$, Rif$^R$ | MB_8630 | This study |
| SA10GΔ*vipA* | SA10G with *aph* cassette in *vipA* gene (HPY11_14100); Kan$^R$ | MB_8552 | This study |
| A1552-Rif$^S$ | A1552 sensitive to Rifampicin (please check Material and Methods for details on construction) | MB_5161 | This study |
| A1552ΔlacZ::FRT-Kan-pheS*-FRT | A1552 with construct ΔlacZ::FRT-Kan-pheS*-FRT; Rif$^R$, cPhe$^S$ | MB_4609 | (Van der Henst *et al.*, 2018) |
| A1552-Rif$^S$Δ*vipA* | A1552-Rif$^S$ with *aph* cassette in *vipA*; Rif$^R$, Kan$^R$ | MB_8553 | This study |

| | | | |
|---|---|---|---|
| SL6Y-vgrG1ΔACD | SL6Y with truncated *vgrG1* through insertion of a stop codon and insertion of an *aph* cassette that replaces the ACD-encoding sequence; $Kan^R$ | MB_8557 | This study |
| SA10G-vgrG1ΔACD | SA10G with truncated *vgrG1* through insertion of a stop codon and insertion of an *aph* cassette that replaces the ACD-encoding sequence; $Kan^R$ | MB_8558 | This study |
| ATCC25872 | *V. cholerae* non-O1 strain (O37); isolated in 1965, Czechoslovakia; intermediate resistant to Strep | MB_276 | Aldová *et al.*, 1968 |
| ATCC25872Δ*vipA* | ATCC25872 deleted for *vipA* (TransFLP); intermediate resistant to Strep | MB_5065 | Van der Henst *et al.*, 2018 |
| ATCC25872 - vgrG1ΔACD | ATCC25872 with truncated *vgrG1* through insertion of a stop codon and insertion of an *aph* cassette that replaces the ACD-encoding sequence; $Kan^R$, intermediate resistant to Strep | MB_8559 | This study |
| W10G-$Rif^R$ | W10G selected for rifampicin resistance | MB_8562 | This study |
| SA3G-$Rif^R$ | SA3G transformed with PCR of *rpoB* gene containing mutation (S531F) that confers rifampicin resistance | MB_8563 | This study |
| SA5Y-$Rif^R$ | SA5Y selected for rifampicin resistance | MB_8564 | This study |
| SL4G-$Rif^R$ | SL4G selected for rifampicin resistance | MB_8565 | This study |
| SL5Y-$Rif^R$ | SL5Y selected for rifampicin resistance | MB_8566 | This study |
| SO5Y-$Rif^R$ | SO5Y selected for rifampicin resistance | MB_8567 | This study |
| L6G-$Rif^R$ | L6G transformed with PCR of *rpoB* gene containing mutation (S531F) that confers rifampicin resistance | MB_8568 | This study |
| SL6Y-$Rif^R$ | SL6Y selected for rifampicin resistance | MB_8569 | This study |
| SP6G-$Rif^R$ | SP6G selected for rifampicin resistance | MB_8570 | This study |
| SP7G-$Rif^R$ | SP7G transformed with PCR of *rpoB* gene containing mutation (S531F) that confers rifampicin resistance | MB_8571 | This study |
| W6G-$Rif^R$ | W6G selected for rifampicin resistance | MB_8572 | This study |
| W7G-$Rif^R$ | W7G selected for rifampicin resistance | MB_8573 | This study |
| E7G-$Rif^R$ | E7G selected for rifampicin resistance | MB_8574 | This study |
| SA7G-$Rif^R$ | SA7G selected for rifampicin resistance | MB_8575 | This study |
| SA10G-$Rif^R$ | SA10G selected for rifampicin resistance | MB_8576 | This study |
| SP7GΔAux6 | SP7G with *aph* cassette in the end of T6SS Aux 6 (HPY08_16370-16380); $Kan^R$ | MB_8631 | This study |
| SP7GΔAux6-$Rif^R$ | SP7G with *aph* cassette in the end of T6SS Aux 6 (HPY08_16370-16380); $Kan^R$; $Rif^R$ | MB_8632 | This study |
| | | | |
| ***E. coli and K. pneumoniae*** | | | |
| TOP 10 | F- mcrA Δ(mrr-hsdRMS-mcrBC) φ80lacZΔM15 ΔlacX74 nupG recA1 araD139 Δ(ara-leu)7697 galE15 galK16 rpsL(StrR) endA1 λ- | MB_741 | This study |
| *K. pneumoniae* | *Klebsiella* sp. / pANT5 (GFP) | MB_6380 | (Benghezal *et al.*, 2006) |

*reference locus tags belong to reference strain N16961 according to Heidelberg *et al.*, 2000.

**Table S2.** Information on long-read whole genome sequencing data and assemblies of NCBI BioProject PRJNA633476.

| Strain name | Strain ID | BioSample | GenBank accession numbers | Number of bases | Number of reads | Mean read length | Total number of contigs | Contig length after circularization | Total genome size | Mean coverage | GC% content |
|---|---|---|---|---|---|---|---|---|---|---|---|
| **W10G** | MB#5537 | SAMN14944057 | CP053794 (chr1) CP053795 (chr2) | 2,050,336,308 bp | 119,162 | 17,206 bp | 2 (chr1+chr2) | 3,033,188 bp (chr1) 1,028,867 bp (chr2) | 4,062,055 bp | 292 x | 47.7% (chr1) 47.2% (chr2) |
| **SA3G** | MB#957 | SAMN14944058 | CP053744 (chr1) CP053745 (chr2) | 1,239,715,817 bp | 70,375 | 17,615 bp | 2 (chr1+chr2) | 3,020,084 bp (chr1) 1,159,698 bp (chr2) | 4,179,782 bp | 201 x | 47.6% (chr1) 47.0% (chr2) |
| **SA5Y#** | MB#353 | SAMN08813238 | CP028892 (chr1) CP028893 (chr2) | 635,540,812 bp | 35,390 | 17,958 bp | 2 (chr1+chr2) | 2,955,400 bp (chr1) 1,095,478 bp (chr2) | 4,050,878 bp | 133 x | 47.8% (chr1) 46.8% (chr2) |
| **SL4G** | MB#955 | SAMN14944065 | CP053796 (chr1) CP053797 (chr2) | 1,083,962,388 bp | 55,665 | 19,472 bp | 2 (chr1+chr2) | 2,954,462 bp (chr1) 1,173,143 bp (chr2) | 4,127,605 bp | 186 x | 47.8% (chr1) 46.4% (chr2) |
| **SL5Y** | MB#954 | SAMN14944067 | CP053798 (chr1) CP053799 (chr2) | 1,623,840,987 bp | 84,121 | 19,303 bp | 2 (chr1+chr2) | 2,947,299 bp (chr1) 1,086,276 bp (chr2) | 4,033,575 bp | 336 x | 47.8% (chr1) 46.9% (chr2) |
| **SO5Y** | MB#960 | SAMN14944069 | CP053800 (chr1) CP053801 (chr2) | 865,600,319 bp | 47,657 | 18,163 bp | 2 (chr1+chr2) | 2,947,270 bp (chr1) 1,049,609 bp (chr2) | 3,996,879 bp | 245 x | 47.9% (chr1) 47.0% (chr2 |
| **L6G** | MB#956 | SAMN14944070 | CP053802 (chr1) CP053803 (chr2) | 1,134,851,816 bp | 70,295 | 16,144 bp | 2 (chr1+chr2) | 2,829,740 bp (chr1) 1,195,206 bp (chr2) | 4,024,946 bp | 225 x | 48.1% (chr1) 46.3% (chr2) |
| **SL6Y** | MB#953 | SAMN14944072 | CP053804 (chr1) CP053805 (chr2) | 826,125,289 bp | 48,156 | 17,155 bp | 2 (chr1+chr2) | 2,900,064 bp (chr1) 1,143,503 bp (chr2) | 4,043,567 bp | 175 x | 47.8% (chr1) 46.7% (chr2) |
| **SP6G** | MB#964 | SAMN14944080 | CP053806 (chr1) CP053807 (chr2) | 874,659,808 bp | 47,923 | 18,251 bp | 2 (chr1+chr2) | 2,947,818 bp (chr1) 1,229,641 bp (chr2) | 4,177,459 bp | 154 x | 47,8% (chr1) 46.7% (chr2) |
| **SP7G** | MB#952 | SAMN14944081 | CP053808 (chr1) CP053809 (chr2) | 1,339,636,117 bp | 73,934 | 18,119 bp | 3 (chr1+chr2) | 2,868,038 bp (chr1) 1,217,051 bp (chr2) | 4,085,089 bp | 217 x | 48.0% (chr1) 46.5% (chr2) |
| **W6G** | MB#354 | SAMN14944082 | CP053810 (chr1) CP053811 (chr2) CP053812 (plasmid) | 1,247,754,152 bp | 69,891 | 17,852 bp | 3 (chr1+chr2+ plasmid) | 2,967,360 bp (chr1) 1,202,132 bp (chr2) 306,484 bp (plas) | 4,475,976 bp | 192 x | 47.8% (chr1) 46.6% (chr2) 42.2% (plas) |
| **W7G** | MB#962 | SAMN14944083 | CP053813 (chr1) | 1,490,677,399 bp | 86,303 | 17,272 bp | 3 (chr1+chr2+ plasmid) | 2,967,793 bp (chr1) 1,202,155 bp (chr2) 306,481 bp (plas) | 4,476,429 bp | 223 x | 47.8% (chr1) 46.6% (chr2) 42.2% (plas) |

| | | | | | | | | | | | |
|---|---|---|---|---|---|---|---|---|---|---|---|
| | | | CP053814 (chr2) CP053815 (plasmid) | | | | | | | | |
| **E7G** | MB#963 | SAMN14944111 | CP053822 (chr1) CP053823 (chr2) CP053824 (plasmid) | 1,477,482,932 bp | 83,856 | 17,619 bp | 3 (chr1+chr2+ plasmid) | 3,040,343 bp (chr1) 1,199,805 bp (chr2) 80,726 bp (plas) | 4,320,874 bp | 194 x | 47.8% (chr1) 46.5% (chr2) 46.3% (plas) |
| **SA7G** | MB#959 | SAMN14944112 | CP053816 (chr1) CP053817 (chr2) CP053818 (plasmid 1) CP053819 (plasmid 2) | 1,689,606,494 bp | 98,160 | 17,212 bp | 4 (chr1+chr2+ plasmid 1 + 2) | 3,004,584 bp (chr1) 1,182,929 bp (chr2) 79,397 bp (plas1) 80,721 bp (plas2) | 4,347,631 bp | 245 x | 47.8% (chr1) 46.5% (chr2) 42.2% (plas1) 46.3% (plas2) |
| **SA10G** | MB#5539 | SAMN14944113 | CP053820 (chr1) CP053821 (chr2) | 1,643,277,942 bp | 91,387 | 17,981 bp | 2 (chr1+chr2) | 3,010,605 bp (chr1) 1,166,701 bp (chr2) | 4,177,306 bp | 269 x | 47.8% (chr1) 46.6% (chr2) |

#Data based on Matthey *et al.*, 2018

**Table S3.** BlastP-predicted T6SS effector and immunity proteins[1] of pandemic, non-pandemic toxigenic, and environmental *V. cholerae* strains.

| Clade | Strain name | Major cluster E/I | Aux cluster 1 *vgrG1* | Aux cluster 1 E/I | Aux cluster 2 E/I | Aux cluster 3 E/I |
|---|---|---|---|---|---|---|
| Pandemic | A1552 | Encoded protein with 1018 aa; evolved VgrG3 with predicted peptidoglycan hydrolase domain; A-type. | Encoded protein with 1164 aa; evolved VgrG1 with an actin crosslinking domain (ACD). | Encoded protein with 642 aa; lipase class 3; A-type. | Encoded protein with 1086 aa; VasX; pore forming activity[2]; A-type. | Encoded protein with 224 aa; papain-like NlpC/P60 peptidase[3]; A-type. |
| D | SA10G* | Encoded protein with 995 aa; evolved VgrG3 with predicted peptidoglycan hydrolase and pesticin-like lysozyme domains; J-type. | Encoded protein with 1164 aa; evolved VgrG1 with an actin crosslinking domain (ACD). | Encoded protein with 710 aa; uncharacterized alpha/beta hydrolase domain (DUF2235); C-type. | Encoded protein with 1194 aa; predicted lysin motif (binds peptidoglycan), but also with pore formation activity[4]; E-type. | Aux cluster 3 is absent. |
| | E7G* | Encoded protein with 995 aa; evolved VgrG3 with predicted peptidoglycan hydrolase and pesticin-like lysozyme domains; J-type. | Encoded protein with 1164 aa; evolved VgrG1 with an actin crosslinking domain (ACD). | Encoded protein with 710 aa; uncharacterized alpha/beta hydrolase domain (DUF2235); C-type. | Encoded protein with 1197 aa; predicted lysin motif (binds peptidoglycan), but also with pore formation activity[4]; E-type. | Aux cluster 3 is absent. |
| | SA7G* | Encoded protein with 995 aa; evolved VgrG3 with predicted peptidoglycan hydrolase and pesticin-like lysozyme domains; J-type. | Encoded protein with 1164 aa; evolved VgrG1 with an actin crosslinking domain (ACD). | Encoded protein with 710 aa; uncharacterized alpha/beta hydrolase domain (DUF2235); C-type. | Encoded protein with 1197 aa; predicted lysin motif (binds peptidoglycan), but also with pore formation activity[4]; E-type. | Aux cluster 3 is absent. |
| C | SL6Y | Encoded protein with 977 aa; evolved VgrG3 with a predicted peptidoglycan hydrolase and phage-like lysozyme domains. E-type. | Encoded protein with 1164 aa; evolved VgrG1 with an actin crosslinking domain (ACD). | Encoded protein with 710 aa; uncharacterized alpha/beta hydrolase domain (DUF2235); C-type. | Encoded protein with 1086 aa; 98.3% identical to A1552's VasX; pore forming activity[2]; A-type. | Aux cluster 3 is absent. |
| | SP6G* | Encoded protein with 1052 aa; evolved VgrG3 with a predicted peptidoglycan hydrolase domain; H-type. | Encoded protein with 1164 aa; evolved VgrG1 with an actin crosslinking domain (ACD). | Encoded protein with 710 aa; uncharacterized alpha/beta hydrolase domain (DUF2235); C-type. | Encoded protein with 1087 aa; 94.8% identical to A1552's VasX; pore forming activity[2]; A-type. | Aux cluster 3 is absent. |
| | L6G* | Encoded protein with 989 aa; evolved VgrG3 with a predicted peptidoglycan hydrolase and lambda phage-like lysozyme domains; K-type. | Encoded protein with 1167 aa; evolved VgrG1 with an actin crosslinking domain (ACD). | Encoded protein with 710 aa; uncharacterized alpha/beta hydrolase domain (DUF2235); C-type. | Encoded protein with 1194 aa; predicted lysin motif (binds peptidoglycan), but also with pore formation activity[4]; E-type. | Aux cluster 3 is absent. |
| | W6G* | Encoded protein with 1052 aa; evolved VgrG3 with a predicted peptidoglycan hydrolase domain; H-type. | Encoded protein with 1164 aa; evolved VgrG1 with an actin crosslinking domain (ACD). | Encoded protein with 710 aa; uncharacterized alpha/beta hydrolase domain (DUF2235); C-type. | Encoded protein with 1088 aa; 94.1% identical to A1552's VasX; pore forming activity[2]; A-type. | Aux cluster 3 is absent. |
| | W7G* | Encoded protein with 1052 aa; evolved VgrG3 with a predicted peptidoglycan hydrolase domain; H-type. | Encoded protein with 1164 aa; evolved VgrG1 with an actin crosslinking domain (ACD). | Encoded protein with 710 aa; uncharacterized alpha/beta hydrolase domain (DUF2235); C-type. | Encoded protein with 1088 aa; 94.1% identical to A1552's VasX; pore forming activity[2]; A-type. | Aux cluster 3 is absent. |

| | | | | | | |
|---|---|---|---|---|---|---|
| | **SP7G**** | Encoded protein with 439 aa, independent from VgrG3 (structural only); cell adhesion function[4]; B-type. | Encoded protein with 1164 aa; evolved VgrG1 with an actin crosslinking domain (ACD). | Encoded protein with 710 aa; uncharacterized alpha/beta hydrolase domain (DUF2235); C-type. | Encoded protein with 1194 aa; predicted lysin motif (binds peptidoglycan), but also with pore formation activity[4]; E-type. | Aux cluster 3 is absent. |
| **A** | **W10G** | Encoded protein with 1018 aa; evolved VgrG3 with predicted peptidoglycan hydrolase domain; A-type. | Encoded protein with 684 aa; structural VgrG1 protein. | PacBio sequencing artifact in the effector gene (HPY12_07370). Region was Sanger sequenced and manually corrected before translation to allow typing. Encoded protein with 710 aa; uncharacterized alpha/beta hydrolase domain (DUF2235); C-type. | PacBio sequencing artifact in the effector gene (HPY12_14010). Region was Sanger sequenced and manually corrected before translation to allow typing. Encoded protein with 1086 aa; VasX; pore forming activity[2]; A-type. | Aux cluster 3 is absent. |
| **B** | **SA3G** | Encoded protein with 1018 aa; evolved VgrG3 with predicted peptidoglycan hydrolase domain; A-type. | Encoded protein with 684 aa; structural VgrG1 protein. | Encoded protein with 710 aa; uncharacterized alpha/beta hydrolase domain (DUF2235); C-type. | Encoded protein with 1087 aa; 94.3% identical to A1552's VasX; pore forming activity[2]; A-type. | Aux cluster 3 is absent. |
| | **SA5Y** | Encoded protein with 995 aa; evolved VgrG3 with predicted peptidoglycan hydrolase and pesticin-like lysozyme domains; J-type. | Encoded protein with 684 aa; structural VgrG1 protein. | Encoded protein with 710 aa; uncharacterized alpha/beta hydrolase domain (DUF2235); C-type. | Encoded protein with 1088 aa; 93.7% identical to A1552's VasX; pore forming activity[2]; A-type. | Aux cluster 3 is absent. |
| | **SL4G*** | Encoded protein with 995 aa; evolved VgrG3 with predicted peptidoglycan hydrolase and pesticin-like lysozyme domains; J-type. | Encoded protein with 684 aa; structural VgrG1 protein. | Encoded protein with 710 aa; uncharacterized alpha/beta hydrolase domain (DUF2235); C-type. | Encoded protein with 1088 aa; 93.7% identical to A1552's VasX; pore forming activity[2]; A-type. | Aux cluster 3 is absent. |
| | **SL5Y*** | Encoded protein with 995 aa; evolved VgrG3 with predicted peptidoglycan hydrolase and pesticin-like lysozyme domains; J-type. | Encoded protein with 684 aa; structural VgrG1 protein. | Encoded protein with 710 aa; uncharacterized alpha/beta hydrolase domain (DUF2235); C-type. | Encoded protein with 1088 aa; 93.7% identical to A1552's VasX; pore forming activity[2]; A-type. | Aux cluster 3 is absent. |
| | **SO5Y*** | Encoded protein with 1022 aa; evolved VgrG3 with predicted peptidoglycan hydrolase and muraidase (N-acetylmuramidase) domains; I-type. | Encoded protein with 684 aa; structural VgrG1 protein. | Encoded protein with 710 aa; uncharacterized alpha/beta hydrolase domain (DUF2235); C-type. | Encoded protein with 1220 aa; predicted lysin motif (binds peptidoglycan); D-type. | Aux cluster 3 is absent. |
| **Non-pandemic** | **ATCC25872** | Encoded protein with 1018 aa; evolved VgrG3 with predicted peptidoglycan hydrolase domain; A-type. | Encoded protein with 1164 aa; evolved VgrG1 with an actin crosslinking domain (ACD). | Encoded protein with 642 aa; lipase class 3; A-type. | Encoded protein with 1086 aa; VasX; pore forming activity[2]; A-type. | Encoded protein with 224 aa; papain-like NlpC/P60 peptidase[3]; A-type. |

[1] Typing according to Unterweger *et al.*, 2014 & Kirchberger *et al.*, 2017.
[2] According to Miyata *et al.*, 2011.
[3] According to Hersch *et al.*, 2020.
[4] According to Unterweger *et al.*, 2014.

* In PGAP annotation, *vgrG3* gene was annotated without the effector portion. Prokka annotation used instead for this study.
** Strain SP7G (clade C) contains an additional T6SS cluster, called Aux 6, which has the same gene order as auxiliary clusters 1 and 2 (see Fig. 4).

**Table S4.** Matrices of % identity (percentage of residues which are identical) among VgrG1-ACD proteins in T6SS auxiliary cluster 1 from clade C and D environmental *V. cholerae* strains and pandemic strain A1552.

| VgrG1-ACD full protein sequence | | | | | | | | | | |
|---|---|---|---|---|---|---|---|---|---|---|
| | A1552 | SA10G | E7G | SA7G | SL6Y | SP6G | L6G | W7G | W6G | SP7G |
| A1552 | | 98.2 | 98.2 | 98.2 | 98.6 | 98.8 | 97.3 | 98.5 | 98.5 | 98.4 |
| SA10G | | | 100 | 100 | 98.9 | 98.9 | 97.2 | 99.1 | 99.1 | 98.9 |
| E7G | | | | 100 | 98.9 | 98.9 | 97.2 | 99.1 | 99.1 | 98.9 |
| SA7G | | | | | 98.9 | 98.9 | 97.2 | 99.1 | 99.1 | 98.9 |
| SL6Y | | | | | | 98.9 | 97.4 | 98.9 | 98.9 | 98.9 |
| SP6G | | | | | | | 97.4 | 99.1 | 99.1 | 99.1 |
| L6G | | | | | | | | 97.7 | 97.7 | 97.4 |
| W7G | | | | | | | | | 100 | 99.4 |
| W6G | | | | | | | | | | 99.4 |
| SP7G | | | | | | | | | | |

| Actin cross-linking domain (ACD) protein sequence only | | | | | | | | | | |
|---|---|---|---|---|---|---|---|---|---|---|
| | A1552 | SA10G | E7G | SA7G | SL6Y | SP6G | L6G | W7G | W6G | SP7G |
| A1552 | | 99.6 | 99.6 | 99.6 | 99.6 | 99.6 | 99.6 | 99.6 | 99.6 | 99.4 |
| SA10G | | | 100 | 100 | 100 | 99.6 | 100 | 100 | 100 | 99.8 |
| E7G | | | | 100 | 100 | 99.6 | 100 | 100 | 100 | 99.8 |
| SA7G | | | | | 100 | 99.6 | 100 | 100 | 100 | 99.8 |
| SL6Y | | | | | | 99.6 | 100 | 100 | 100 | 99.8 |
| SP6G | | | | | | | 99.6 | 99.6 | 99.6 | 99.4 |
| L6G | | | | | | | | 100 | 100 | 99.8 |
| W7G | | | | | | | | | 100 | 99.8 |
| W6G | | | | | | | | | | 99.8 |
| SP7G | | | | | | | | | | |

Green: 100% identity
Yellow: 90% - 99.99% identity

**Table S5.** Matrices of % identity (percentage of residues which are identical) among effector and immunity proteins harbored in the T6SS large cluster from environmental *V. cholerae* strains and pandemic strain A1552.

**Large cluster – Effector**

| | A1552 | SA10G | E7G | SA7G | SL6Y | SP6G | L6G | W7G | W6G | SP7G | W10G | SA3G | SA5Y | SL4G | SL5Y | SO5Y |
|---|---|---|---|---|---|---|---|---|---|---|---|---|---|---|---|---|
| A1552 | | 8.9 | 8.9 | 8.9 | 7.5 | 6.7 | 5.7 | 6.7 | 6.7 | 5.3 | 100 | 99.5 | 8.9 | 8.8 | 8.9 | 9.8 |
| SA10G | | | 100 | 100 | 13.9 | 8.4 | 8.2 | 8.4 | 8.4 | 7.4 | 8.9 | 8.9 | 98.9 | 98.9 | 98.9 | 10.4 |
| E7G | | | | 100 | 13.9 | 8.4 | 8.2 | 8.4 | 8.4 | 7.4 | 8.9 | 8.9 | 98.9 | 98.9 | 98.9 | 10.4 |
| SA7G | | | | | 13.9 | 8.4 | 8.2 | 8.4 | 8.4 | 7.4 | 8.9 | 8.9 | 98.9 | 98.9 | 98.9 | 10.4 |
| SL6Y | | | | | | 8.6 | 10.6 | 8.6 | 8.6 | 5.6 | 7.5 | 7.5 | 13.8 | 13.8 | 13.8 | 6.5 |
| SP6G | | | | | | | 9.2 | 100 | 100 | 5.7 | 6.7 | 6.7 | 8.3 | 8.3 | 8.4 | 10.8 |
| L6G | | | | | | | | 9.2 | 9.2 | 5.4 | 5.7 | 5.7 | 8.7 | 8.6 | 8.4 | 8.5 |
| W7G | | | | | | | | | 100 | 5.7 | 6.7 | 6.7 | 8.3 | 8.3 | 8.4 | 10.8 |
| W6G | | | | | | | | | | 5.7 | 6.7 | 6.7 | 8.3 | 8.3 | 8.4 | 10.8 |
| SP7G | | | | | | | | | | | 5.3 | 5.3 | 7.6 | 7.6 | 7.4 | 5.3 |
| W10G | | | | | | | | | | | | 99.5 | 8.9 | 8.8 | 8.9 | 9.8 |
| SA3G | | | | | | | | | | | | | 8.9 | 8.8 | 8.9 | 9.8 |
| SA5Y | | | | | | | | | | | | | | 100 | 100 | 10.8 |
| SL4G | | | | | | | | | | | | | | | 100 | 10.8 |
| SL5Y | | | | | | | | | | | | | | | | 10.9 |
| SO5Y | | | | | | | | | | | | | | | | |

**Large cluster – Immunity**

| | A1552 | SA10G | E7G | SA7G | SL6Y | SP6G | L6G | W7G | W6G | SP7G | W10G | SA3G | SA5Y | SL4G | SL5Y | SO5Y |
|---|---|---|---|---|---|---|---|---|---|---|---|---|---|---|---|---|
| A1552 | | 11.7 | 11.7 | 11.7 | 7.7 | 8.1 | 12.0 | 8.1 | 8.1 | 6.4 | 100 | 100 | 11.7 | 11.7 | 11.7 | 15.8 |
| SA10G | | | 100 | 100 | 10.8 | 6.8 | 8.4 | 6.8 | 6.8 | 10.9 | 11.7 | 11.7 | 100 | 100 | 100 | 10.5 |
| E7G | | | | 100 | 10.8 | 6.8 | 8.4 | 6.8 | 6.8 | 10.9 | 11.7 | 11.7 | 100 | 100 | 100 | 10.5 |
| SA7G | | | | | 10.8 | 6.8 | 8.4 | 6.8 | 6.8 | 10.9 | 11.7 | 11.7 | 100 | 100 | 100 | 10.5 |
| SL6Y | | | | | | 8.5 | 15.8 | 8.5 | 8.5 | 8.8 | 7.7 | 7.7 | 10.8 | 10.8 | 10.8 | 10.1 |
| SP6G | | | | | | | 7.0 | 100 | 100 | 8.4 | 8.1 | 8.1 | 6.8 | 6.8 | 6.8 | 9.7 |
| L6G | | | | | | | | 7.0 | 7.0 | 8.5 | 12.0 | 12.0 | 8.4 | 8.4 | 8.4 | 11.2 |
| W7G | | | | | | | | | 100 | 8.4 | 8.1 | 8.1 | 6.8 | 6.8 | 6.8 | 9.7 |
| W6G | | | | | | | | | | 8.4 | 8.1 | 8.1 | 6.8 | 6.8 | 6.8 | 9.7 |
| SP7G | | | | | | | | | | | 6.4 | 6.4 | 10.9 | 10.9 | 10.9 | 7.5 |
| W10G | | | | | | | | | | | | 100 | 11.7 | 11.7 | 11.7 | 15.8 |
| SA3G | | | | | | | | | | | | | 11.7 | 11.7 | 11.7 | 15.8 |
| SA5Y | | | | | | | | | | | | | | 100 | 100 | 10.5 |
| SL4G | | | | | | | | | | | | | | | 100 | 10.5 |
| SL5Y | | | | | | | | | | | | | | | | 10.5 |
| SO5Y | | | | | | | | | | | | | | | | |

Green: 100% identity (same family and subfamily)
Yellow: 90% - 99.99% identity (same family, with some polymorphism)
Orange: 30% - 90% identity (same family, with considerable polymorphism)
Red: <30% identity (different families)

**Table S6.** Matrices of % identity (percentage of residues which are identical) among effector and immunity proteins harbored in the T6SS auxiliary cluster 1 from environmental *V. cholerae* strains and pandemic strain A1552.

**Auxiliary cluster 1 – Effector**

| | A1552 | SA10G | E7G | SA7G | SL6Y | SP6G | L6G | W7G | W6G | SP7G | W10G | SA3G | SA5Y | SL4G | SL5Y | SO5Y |
|---|---|---|---|---|---|---|---|---|---|---|---|---|---|---|---|---|
| A1552 | | 15.0 | 15.0 | 15.0 | 15.5 | 15.0 | 15.5 | 15.8 | 15.8 | 15.8 | 14.6 | 15.9 | 15.9 | 15.9 | 14.9 | 15.3 |
| SA10G | | | 100 | 100 | 90.4 | 99.7 | 90.6 | 94.6 | 94.6 | 94.6 | 91.3 | 94.5 | 94.5 | 94.4 | 92.5 | 98.9 |
| E7G | | | | 100 | 90.4 | 99.7 | 90.6 | 94.6 | 94.6 | 94.6 | 91.3 | 94.5 | 94.5 | 94.4 | 92.5 | 98.9 |
| SA7G | | | | | 90.4 | 99.7 | 90.6 | 94.6 | 94.6 | 94.6 | 91.3 | 94.5 | 94.5 | 94.4 | 92.5 | 98.9 |
| SL6Y | | | | | | 90.4 | 99.9 | 92.9 | 92.9 | 92.9 | 87.0 | 92.8 | 92.8 | 92.7 | 94.5 | 91.1 |
| SP6G | | | | | | | 90.6 | 94.4 | 94.4 | 94.3 | 91.3 | 94.2 | 94.2 | 94.1 | 92.3 | 98.9 |
| L6G | | | | | | | | 93.1 | 93.1 | 93.1 | 87.2 | 92.9 | 92.9 | 92.8 | 94.6 | 91.3 |
| W7G | | | | | | | | | 100 | 100 | 90.3 | 99.6 | 99.6 | 99.4 | 91.0 | 94.6 |
| W6G | | | | | | | | | | 100 | 90.3 | 99.6 | 99.6 | 99.4 | 91.0 | 94.6 |
| SP7G | | | | | | | | | | | 90.3 | 99.6 | 99.6 | 99.4 | 91.0 | 94.6 |
| W10G | | | | | | | | | | | | 90.1 | 90.1 | 89.9 | 89.9 | 91.9 |
| SA3G | | | | | | | | | | | | | 100 | 99.9 | 90.8 | 94.5 |
| SA5Y | | | | | | | | | | | | | | 99.9 | 90.83 | 94.5 |
| SL4G | | | | | | | | | | | | | | | 90.7 | 94.4 |
| SL5Y | | | | | | | | | | | | | | | | 92.7 |
| SO5Y | | | | | | | | | | | | | | | | |

**Auxiliary cluster 1 – Immunity**

| | A1552 | SA10G | E7G | SA7G | SL6Y | SP6G | L6G | W7G | W6G | SP7G | W10G | SA3G | SA5Y | SL4G | SL5Y | SO5Y |
|---|---|---|---|---|---|---|---|---|---|---|---|---|---|---|---|---|
| A1552 | | 15.5 | 15.5 | 15.5 | 15.2 | 15.5 | 15.2 | 15.9 | 15.9 | 15.9 | 14.0 | 15.9 | 15.9 | 15.9 | 15.1 | 15.1 |
| SA10G | | | 100 | 100 | 72.1 | 99.6 | 71.7 | 85.7 | 85.7 | 85.7 | 78.3 | 85.7 | 85.7 | 85.7 | 76.2 | 97.5 |
| E7G | | | | 100 | 72.1 | 99.6 | 71.7 | 85.7 | 85.7 | 85.7 | 78.3 | 85.7 | 85.7 | 85.7 | 76.2 | 97.5 |
| SA7G | | | | | 72.1 | 99.6 | 71.7 | 85.7 | 85.7 | 85.7 | 78.3 | 85.7 | 85.7 | 85.7 | 76.2 | 97.5 |
| SL6Y | | | | | | 72.1 | 99.6 | 78.7 | 78.7 | 78.7 | 65.6 | 78.7 | 78.7 | 78.7 | 91.4 | 72.5 |
| SP6G | | | | | | | 71.7 | 85.7 | 85.7 | 85.7 | 77.9 | 85.7 | 85.7 | 85.7 | 75.8 | 97.1 |
| L6G | | | | | | | | 78.3 | 78.3 | 78.3 | 65.2 | 78.3 | 78.3 | 78.3 | 90.9 | 72.1 |
| W7G | | | | | | | | | 100 | 100 | 71.3 | 100 | 100 | 100 | 73.0 | 85.2 |
| W6G | | | | | | | | | | 100 | 71.3 | 100 | 100 | 100 | 73.0 | 85.2 |
| SP7G | | | | | | | | | | | 71.3 | 100 | 100 | 100 | 73.0 | 85.2 |
| W10G | | | | | | | | | | | | 71.3 | 71.3 | 71.3 | 70.2 | 79.2 |
| SA3G | | | | | | | | | | | | | 100 | 100 | 73.0 | 85.2 |
| SA5Y | | | | | | | | | | | | | | 100 | 73.0 | 85.2 |
| SL4G | | | | | | | | | | | | | | | 73.0 | 85.2 |
| SL5Y | | | | | | | | | | | | | | | | 76.3 |
| SO5Y | | | | | | | | | | | | | | | | |

Green: 100% identity (same family and subfamily)
Yellow: 90% - 99.99% identity (same family, with some polymorphism)
Orange: 30% - 90% identity (same family, with considerable polymorphism)
Red: <30% identity (different families)

**Table S7.** Matrices of % identity (percentage of residues which are identical) among effector and immunity proteins harbored in the T6SS auxiliary cluster 2 from environmental *V. cholerae* strains and pandemic strain A1552.

**Auxiliary cluster 2 – Effector**

| | A1552 | SA10G | E7G | SA7G | SL6Y | SP6G | L6G | W7G | W6G | SP7G | W10G | SA3G | SA5Y | SL4G | SL5Y | SO5Y |
|---|---|---|---|---|---|---|---|---|---|---|---|---|---|---|---|---|
| A1552 | | 14.6 | 14.4 | 14.4 | 98.3 | 94.8 | 14.8 | 94.0 | 94.0 | 14.8 | 96.5 | 94.3 | 93.6 | 93.6 | 93.6 | 12.7 |
| SA10G | | | 95.2 | 95.2 | 14.5 | 15.3 | 97.6 | 15.2 | 15.2 | 97.5 | 14.9 | 15.0 | 15.2 | 15.2 | 15.2 | 20.2 |
| E7G | | | | 100 | 14.3 | 15.0 | 95.1 | 15.0 | 15.0 | 95.0 | 14.6 | 14.7 | 15.1 | 15.1 | 15.1 | 19.8 |
| SA7G | | | | | 14.3 | 15.0 | 95.1 | 15.0 | 15.0 | 95.0 | 14.6 | 14.7 | 15.1 | 15.1 | 15.1 | 19.8 |
| SL6Y | | | | | | 94.1 | 14.7 | 93.7 | 93.5 | 14.7 | 95.7 | 93.8 | 93.7 | 93.7 | 93.7 | 12.8 |
| SP6G | | | | | | | 15.4 | 96.1 | 96.1 | 15.4 | 94.7 | 98.8 | 95.0 | 95.0 | 95.0 | 12.7 |
| L6G | | | | | | | | 15.3 | 15.3 | 99.9 | 15.0 | 15.2 | 15.4 | 15.4 | 15.4 | 19.8 |
| W7G | | | | | | | | | 100 | 15.3 | 93.8 | 94.8 | 99.0 | 99.0 | 99.0 | 13.0 |
| W6G | | | | | | | | | | 15.3 | 93.8 | 94.8 | 99.0 | 99.0 | 99.0 | 13.0 |
| SP7G | | | | | | | | | | | 15.0 | 15.2 | 15.4 | 15.4 | 15.4 | 19.8 |
| W10G | | | | | | | | | | | | 94.0 | 93.4 | 93.4 | 93.4 | 12.8 |
| SA3G | | | | | | | | | | | | | 94.0 | 94.0 | 94.0 | 12.4 |
| SA5Y | | | | | | | | | | | | | | 100 | 100 | 13.0 |
| SL4G | | | | | | | | | | | | | | | 100 | 13.0 |
| SL5Y | | | | | | | | | | | | | | | | 13.0 |
| SO5Y | | | | | | | | | | | | | | | | |

**Auxiliary cluster 2 – Immunity**

| | A1552 | SA10G | E7G | SA7G | SL6Y | SP6G | L6G | W7G | W6G | SP7G | W10G | SA3G | SA5Y | SL4G | SL5Y | SO5Y |
|---|---|---|---|---|---|---|---|---|---|---|---|---|---|---|---|---|
| A1552 | | 13.2 | 12.9 | 12.9 | 99.6 | 72.3 | 12.9 | 68.7 | 68.7 | 12.9 | 83.9 | 71.9 | 68.3 | 68.3 | 68.2 | 12.4 |
| SA10G | | | 73.1 | 73.1 | 13.2 | 13.6 | 92.5 | 12.9 | 12.9 | 92.2 | 12.5 | 13.6 | 12.9 | 12.9 | 12.9 | 9.4 |
| E7G | | | | 100 | 12.9 | 11.2 | 73.9 | 11.9 | 11.9 | 73.9 | 12.2 | 11.5 | 11.9 | 11.9 | 11.9 | 8.1 |
| SA7G | | | | | 12.9 | 11.2 | 73.9 | 11.9 | 11.9 | 73.9 | 12.2 | 11.5 | 11.9 | 11.9 | 11.9 | 8.1 |
| SL6Y | | | | | | 71.9 | 12.9 | 68.3 | 68.3 | 12.9 | 83.5 | 71.5 | 67.9 | 67.9 | 67.9 | 12.4 |
| SP6G | | | | | | | 12.5 | 80.1 | 80.1 | 12.5 | 74.0 | 99.6 | 80.5 | 80.5 | 80.5 | 12.1 |
| L6G | | | | | | | | 12.5 | 12.5 | 99.7 | 12.2 | 12.5 | 12.5 | 12.5 | 12.5 | 9.9 |
| W7G | | | | | | | | | 100 | 12.5 | 67.5 | 79.7 | 99.2 | 99.2 | 99.2 | 11.0 |
| W6G | | | | | | | | | | 12.5 | 67.5 | 79.7 | 99.2 | 99.2 | 99.2 | 11.0 |
| SP7G | | | | | | | | | | | 12.2 | 12.5 | 12.5 | 12.5 | 12.5 | 9.9 |
| W10G | | | | | | | | | | | | 73.6 | 67.5 | 67.5 | 67.5 | 11.2 |
| SA3G | | | | | | | | | | | | | 80.1 | 80.1 | 80.1 | 12.1 |
| SA5Y | | | | | | | | | | | | | | 100 | 100 | 11.0 |
| SL4G | | | | | | | | | | | | | | | 100 | 11.0 |
| SL5Y | | | | | | | | | | | | | | | | 11.0 |
| SO5Y | | | | | | | | | | | | | | | | |

Green: 100% identity (same family and subfamily)
Yellow: 90% - 99.99% identity (same family, with some polymorphism)
Orange: 30% - 90% identity (same family, with considerable polymorphism)
Red: <30% identity (different families)

## Supporting References


Aldova, E., Laznickova, K., Stepankova, E., and Lietava, J. (1968) Isolation of nonagglutinable vibrios from an enteritis outbreak in Czechoslovakia. *J Infect Dis* **118**: 25–31.

Benghezal, M., Fauvarque, M.O., Tournebize, R., Froquet, R., Marchetti, A., Bergeret, E., *et al.* (2006) Specific host genes required for the killing of *Klebsiella* bacteria by phagocytes. *Cell Microbiol* **8**: 139–148.

Heidelberg, J.F., Elsen, J.A., Nelson, W.C., Clayton, R.A., Gwinn, M.L., Dodson, R.J., *et al.* (2000) DNA sequence of both chromosomes of the cholera pathogen *Vibrio cholerae*. *Nature* **406**: 477–483.

Hersch, S.J., Watanabe, N., Stietz, M.S., Manera, K., Kamal, F., Burkinshaw, B., *et al.* (2020) Envelope stress responses defend against type six secretion system attacks independently of immunity proteins. *Nat Microbiol* **5:** 706–714.

Keymer, D.P., Miller, M.C., Schoolnik, G.K., and Boehm, A.B. (2007) Genomic and phenotypic diversity of coastal *Vibrio cholerae* strains is linked to environmental factors. *Appl Environ Microbiol* **73**: 3705–3714.

Kirchberger, P.C., Unterweger, D., Provenzano, D., Pukatzki, S., and Boucher, Y. (2017) Sequential displacement of type VI secretion system effector genes leads to evolution of diverse immunity gene arrays in *Vibrio cholerae*. *Sci Rep* **7**: 45133.

Matthey, N., Drebes Dörr, N.C., and Blokesch, M. (2018) Long-read-based genome sequences of pandemic and environmental *Vibrio cholerae* strains. *Microbiol Resour Announc* **7**: e01574-18.

Miyata, S.T., Kitaoka, M., Brooks, T.M., McAuley, S.B., and Pukatzki, S. (2011) *Vibrio cholerae* requires the type VI secretion system virulence factor vasx to kill *Dictyostelium discoideum*. *Infect Immun* **79**: 2941–2949.

Unterweger, D., Miyata, S.T., Bachmann, V., Brooks, T.M., Mullins, T., Kostiuk, B., *et al.* (2014) The *Vibrio cholerae* type VI secretion system employs diverse effector modules for intraspecific competition. *Nat Commun* **5**: 3549.

Van der Henst, C., Vanhove, A.S., Drebes Dörr, N.C., Stutzmann, S., Stoudmann, C., Clerc, S., *et al.* (2018) Molecular insights into *Vibrio cholerae*'s intra-amoebal host-pathogen interactions. *Nat Commun* **9**: 3420.

Yildiz, F.H. and Schoolnik, G.K. (1998) Role of *rpoS* in stress survival and virulence of *Vibrio cholerae*. *J Bacteriol* **180**: 773–784.
